# A Population-based Prospective Study to Identify Contributors to Mother and Child Health in Suburban Communities: The Cohort Profile

**Published:** 2018-03

**Authors:** Kourosh HOLAKOUIE-NAIENI, Shahrzad NEMATOLLAHI, Mohammad Ali MANSOURNIA, Mohammad SHEKARI, Teimour AGHA-MOLAYI, Azin ALAVI, Abdul-Hussain MADANI, Nadereh HOLAKOUIE-NAIENI, Hossein SHABKHIZ, Elham TORABI, Hasan AHMADI-GHARAEE

**Affiliations:** 1. Dept. of Epidemiology and Biostatistics, School of Public Health, Tehran University of Medical Sciences, Tehran, Iran; 2. Bandar Abbas Health Research Station, WHO Regional Malaria Training Center, Tehran University of Medical Sciences, Bandar Abbas, Iran; 3. Dept. of Medical Genetics, School of Medicine, Hormozgan University of Medical Sciences, Bandar Abbas, Iran; 4. Dept. of Public Health, School of Public Health, Hormozgan University of Medical Sciences, Bandar Abbas, Iran; 5. Dept. of Obstetrics and Gynecology, School of Medicine, Hormozgan University of Medical Sciences, Bandar Abbas, Iran

**Keywords:** Cohort studies, Suburbs, Pregnancy, Child development, Community assessment project

## Abstract

**Background:**

Following community health assessment Project (CHAP) in suburbs of Bandar Abbas city, health problems in women and children such as pregnancy complications and infant/child impaired growth are highly prevalent. Therefore, the present population-based prospective cohort study investigated the effects of a wide range of modifiable exposures during pregnancy and postpartum on mother and child health.

**Methods:**

The sample comprised of 1000 pregnant women in their first gestational trimester, who live in the three most socially and economically vulnerable neighborhoods of Bandar Abbas, are under recruitment during Feb 2016–18. Four structured questionnaires are being carried out from pregnancy to 30 d, 6 months, and 12 months postpartum. Biologic and ultrasound results are also gathered through hospital and health center records. The study is currently close to the end of the recruitment phase.

**Conclusion:**

The results of the interim and final analyses are being translated into applicable preventive action plans aiming to reduce and control modifiable risk factors for ill-health in mothers and children in suburb communities in South of Iran.

## Background

Nowadays, prioritizing health problems is based on community needs, which underpins problem identification methods to be context-specific. Community Health Assessment Project (CHAP) is a useful tool to recognize community problems and evaluate effectiveness of public health interventions (1). With community cooperation, CHAP analyzes and disseminates community information (2); utilized to assign resources and to gauge evidence-based action plans also known as “special healthcare packages”. CHAP has been running since 2013 in Bandar Abbas city, the capital of Hormozgan Province with 680366 residents. Results of the recent CHAPs (data unpublished) consistent with other research in Bandar Abbas showed that health concerns of the community were mainly problems related to women and children including high-risk pregnancies, low birth weight, childhood malnutrition, and impaired child growth (3–5). Fetal growth restriction remains a major public health concern in developing countries, especially among under-privileged populations (6). The growth restriction not only accelerates Low Birth Weight (LBW: defined as birthweight below 2500 grams (7)); but also contributes to child impaired growth in later life (8).

LBW is associated with mortality and health morbidities (metabolic disorders, or cardiovascular diseases) of the newborn (9, 10). It also contributes to behavioral deficits (educational retardation) (11, 12), malnutrition, and growth retardation in school ages (13). LBW is the main concern in Iran since 8% of infants (a range between 5%–8%) born in 2014 in Iran were LBWs (14). While fetal growth trajectories are attributed to overweight or obesity, the prevalence of under-weight in school-aged children in an underprivileged region of the country is almost one-quarter (15). Fetal restriction is a result of a complex constellation of factors in pre-conception and pregnancy periods. Dietary intakes of iron and vitamin and micronutrients such as caffeine in mother prior and during pregnancy might influence intrauterine growth and the risk of later morbidities in the offspring such as bone fractures, asthma, and allergies (16–19).

Similar to the adverse effects of cigarette smoking, hookah smoking, which has become popular among pregnant women in Iran (20) can increase the risk of low birth weight (21, 22). Furthermore, maternal tobacco smoking during pregnancy is identified as an important risk factor for childhood obesity (23). Other factors such as teenage pregnancy, stressful occupations, chronic conditions such as hypertension, obstetrics problems such as history of stillbirth, insufficient prenatal care, and maternal emotional ill-health can also contribute to fetal growth retardation (6, 24, 25). The role of aforementioned factors can be strengthened in low socio-economic settings. LBW is a well-established key predictor of health inequalities and poverty, and an implication of inadequate health care provision for the lowest socioeconomic positions (6, 7). Pregnant mothers in deprived communities are more likely to have modifiable risk factors of LBW such as malnutrition, physical morbidities (like anemia), insufficient medical care, drug and tobacco use, and obstetrics problems (26–28).

Preventive strategies for growth retardation are most effective when applied within the first two-three years postnatal life (29), but our ability to intervene is limited because we lack a comprehensive understanding of risk and protective factors on this issue. Our information in Iran is even more incomplete. In-depth description of community-specific risk and protective factors that are related to early child development requires studies with large and representative samples, which motivated us to design the present population-based prospective cohort study.

The aim was to investigate the effect of modifiable perinatal and antenatal risk factors on fetal and child growth in three most socially and economically vulnerable populations. Moreover, we intend to measure the following effects on infant and child growth: Gestational nutrition status with emphasize on caffeine intake and dietary supplementation (16, 30); Tobacco smoking and second-hand exposure (22), Maternal mental health during pregnancy (31), Maternal Social capital (31, 32), and Perinatal and antenatal care quality (33).

## Methods

### Time period of the study

The recruitment phase of the study was launched on Feb 2016 and will end on Feb 2018.

The protocol is being conducted by “Bandar Abbas health-Research station” affiliated to Tehran University of Medical Sciences (TUMS); and Bandar Abbas University of Medical Sciences. Consequent phases of the study are also planned to conduct on 1, 6, and 12 months postpartum.

The city of Bandar Abbas is located in the South of Iran and is the capital of Hormozgan Province, an unindustrialized region with a Human Development Index (HDI) of 0.704 in 2013, which ranked 17 among 31 provinces in Iran (34). Its population was 680366 inhabitants in 2016.

### Protocol design and population

The reference population consists of pregnant women residing in the three most socially and economically vulnerable neighborhoods of Bandar Abbas city (namely *Chahestaniha, Do-hezar,* and *Derakht-e-sabz*) regardless of receiving prenatal care at public or private health services. The sample size is estimated to be 1000 pregnant women. Despite the better efficiency and representativeness of “proportional to size” sampling technique (35), due to unavailability of primary Sampling Unites (PSUs, i.e. the population frame of all pregnant women residing in the study areas), the sample was drawn using door-by-door sampling method to retain representativeness (36).

Pregnant women are invited for the interview during pregnancy as the recruitment phase (first phase) of the study. Three consecutive interviews will be followed in 30 d, 6, and 12 months post-partum. On some occasions, hospital records will be monitored to acquire needed information in the phases.

### Inclusion criteria

The inclusion criteria include any pregnant woman who resides in the study area and intends to do so at least by the time of labor. The last criterion is included since majority of the residents in the study areas are migrants with high movement rates. We are excluding non-Iranian women with medically induced pregnancy, or being unable to speak Farsi. The latter criterion was set because of substantial differences in the exposure profiles of pregnant women belonging to minority groups (37).

### Data collection and recruitment strategies

The cohort is still in the recruitment phase. The registration form contains identification data (name, postal address, personal contact information), center where prenatal care visits were performed, and gestational age calculated by ultrasound. The interviews are carried out in the subject’s home, where due to social and cultural restrictions; she will feel more comfortable. The follow-up visits are done by the same manner or by telephone calls as an alternative. We have trained two local females who are healthy volunteers to perform the interviews. The project is further introduced in local media, mosques, and community halls. After filling out the questionnaires, the subject will receive a registration card with the current and future date and time for interviews. They will also receive two free visits to obstetrician and pediatrics in Imam Khomeini general hospital in Bandar Abbas.

### Instruments and variables

The data are gathered using four structured questionnaires in pregnancy, 1, 6 and 12 months postpartum. With slight changes at each phase, the following information is consistently collected at all phases: i) general information of the household, and mother’s obstetrics, reproductive, and medical history; and ii) Maternal lifestyle characteristics including dietary habits, tobacco smoking, and prenatal care. A team of obstetrician, dietician, physician, and epidemiologist checked the validity of the questionnaires. The reliability was assessed using Cronbach’s alpha on a subsample of 30, which yielded 0.69 for dietary habits section and 0.81 for tobacco smoking section. iii) Maternal mental health is consistently measured by Iranian versions of Self-rated health (38), Depression-Anxiety-Stress Scale 21-Items (DASS-21) (39), Social capital questionnaire (40), and 12-items General Health Questionnaire (GHQ-12) (41). Stressful life events during pregnancy and pre-conception (42) and social capital of close contacts (43) after delivery are also gathered.

Socio-economic status (SES) of the household are consistently measured based on nine questions about household possession of private automobile, private motorcycles, refrigerator, dishwashing machine, microwave, personal computer, washing machine, electronic vacuum, and LED/LCD television (44).

Moreover, for those subjects with records of district health center, the information is collected for the second phase. According to the “integrated prenatal care” guideline for healthcare centers, blood tests are routinely requested for pregnant women at the first and second gestational trimesters for examination of CBC, BG, Rh, FBS, HBsAg, and HIV. Urine test (U/A, BUN, and Creatinine) is also routinely requested at the first trimester. Other information such as immunization history, blood pressure, vaginal examinations, and ultrasound scan results are collected and integrated into the cohort dataset. In case of miscarriage, the information will be gathered either from hospital or from mother. The fourth questionnaire contains information regarding neurological and physical development of the child at the age of 12 months, any history of hospitalization and diseases, immunization status, and physical and emotional wellbeing of the mother. The validity of this questionnaire is vetting by a team of experts, and the reliability is to be checked by a subsample of mothers with a 12-month-old child (Table 1).

**Table 1:** Data collected at each stage of the prospective study to identify contributors to mother and child health in suburban communities

	***Pregnancy***	***Birth***	***6 months***	***12 months***
Socio-demographics				
Maternal Age	•	•	•	•
Maternal Education	•	•	•	•
Maternal occupation /employment	•	•	•	•
Current marital status	•	•	•	•
Spouse Age	•	•	•	•
Spouse Educational attainment	•	•	•	•
Spouse occupation	•	•	•	•
Type of family	•	•	•	•
Family income	•	•	•	•
Family assets	•			•
Family monthly expenses (rent, food, healthcare)	•		•	•
Number of available rooms	•	•	•	•
Maternal Mental health status				
Self-rated health	•	•	•	•
Depression	•			•
Anxiety	•			•
Stress	•			•
Social capital			•	•
Obstetrics History				
Time lag of past Deliveries	•	•		
History of Pregnancy complications	•			
History of Birth complications	•			
Current pregnancy				
Menstrual cycle and LMP	•			
Food supplements intake	•	•		
Caffeine intake	•	•		
Alcohol intake	•	•		
Cigarette/ water-pipe/ and substance use	•	•		
History of contraceptive use	•			
Unwanted pregnancy	•			
Medication intake	•	•	•	•
Prenatal care/ Delivery mode		•		
Pregnancy weight gain		•		
Insurance		•	•	•
Vaccination status	•			
Prenatal Stressful Life Events (PSLEs)		•		
Maternal clinical history				
History of communicable and non-communicable diseases	•	•	•	•
Family medical history	•			
Physical examinations				
Weight/height	•	•	•	•
Ultrasound scan	•			
Biologic samples				
Blood	•			
Urine	•			
Infant/ child				
Birth weight/ height/ head circumference		•		
Birth abnormality		•	•	
Childcare		•	•	•
Growth monitoring		•	•	•
Supplement use		•	•	•
Milk or formula feeding		•	•	•
Introduction of foods		•	•	•
Eating behavior/dietary restraint		•	•	•
Illnesses		•	•	•
Medication intake		•	•	•
Hospitalization history		•	•	•
Sleeping arrangements		•	•	•
Sleep/activity		•	•	•
Immunizations		•	•	•

### Statistical analyses

The primary outcome is low birth weight, while subsequent outcome during the second and third phases is impaired growth of child at the ages of 6, and 12 months based on growth monitoring charts. Principal Component Analysis (PCA) is applied for the index of Socio-Economic Status (SES) based on the nine assets listed above. PCA is a multivariate statistical technique that reduces the number of possibly correlated variables and generates fewer uncorrelated variables called principal components (45). The effect of different gestational exposures will be estimated by Generalized Linear Models (GLMs) providing estimate of the risk ratios (RRs) (46). Assessment of additive interactions between each pair of the main exposure variables (i.e. DASS scores, caffeine dosage, water-pipe smoking categories, and social capital score) on the risk of the outcomes will be carried out using Relative Risk due to Interaction (RERI), and Attributable Proportion due to interaction (AP)(47). Longitudinal changes in the effects will be assessed using change score and GEE (Generalized Estimation Equations) models.

### Sample design and statistical power

The prevalence of the explanatory variables ranged from 15% to 50%. Thus, considering a 10% LBW rate (as the initial outcome), the sample size calculated as 1000 pregnant women, with a 5% probability of type I error and a 80% power to detect a risk ratio of 2.6 associated with a prevalence as low as 10% of the hookah smoking in pregnant women(as the least prevalent exposure).

### Data processing

Data is entered in duplicate using the Microsoft Office Excel 2013. The two sets of entries are compared and inconsistencies are being corrected. One person from the investigation team checks the data entry into STATA statistical software. Frequency tables are used to find any inconsistency or mistyping.

### Ethics approval

The Research Ethics Committee of “Ministry of Health and medical education” and “National Institute for Medical Research Development (NIMAD)” approved the protocol (Protocol N. 943607 and ethical N. IR.NIMAD.REC.1396.205). Moreover, ethics committee of school of public health of Tehran University of Medical Sciences (TUMS) with the code of 42933244/3246 approved this research. The mothers who agree to participate will provide written informed consent and will be informed that they could drop out at any time during the study with no harm to themselves or their babies. Due to cultural situations in the area, verbal consent will be sought from the husband, if necessary.

## Discussion

The proposed protocol focuses on the question of child impaired growth, whose rates in developing countries have continued to be increased (7). Despite the decreasing trends of maternal and neonatal morbidity during recent decades in Iran (48), there is still scarce literature hypothesizing the mechanisms of action for modifiable risk factors.

The present protocol aims to determine the effects of individual-level (lifestyle behaviors, mental and physical well-being), and environmental-level (underprivileged neighborhood and social network) factors on mother/child health in South of Iran. The classical risk factors for maternal and neonatal health are only partially explanatory; therefore, we are interested in new hypotheses explaining the magnitude and direction of interactive effects of the risk factors. In order to propose effective action plans, measures of additive interaction are selected to provide public health impact of the effective factors (47). The study will identify risk factors of mother and child suboptimal health status in three most socially and economically vulnerable neighborhoods of Bandar Abbas city. Following identification of the risk factors, the final objective of the study is to develop action plans. This is also the last phase of the CHAP, where health concerns of the community are addressed by evidence-based interventions.

Action plans also have the potency to translate into community-level and national-level health policies according to WHO recommendations for 2025 maternal and child health. Specifically, national-level policies may include interventions to empower and increase educational attainments in women, improvement in social capital for women, and improvement in facility-based peri-natal care in regions with low coverage. Furthermore, community-level policies may include promotion of programs to quit tobacco smoking, provision of intermittent dietary supplements, and improved nutritional recommendations especially for teenaged mothers, and follow-up protocols for children with growth retardation and malnutrition (7).

## Conclusion

The results of the present project will be translated into applicable preventive action plans. These culturally-adjusted action plans aim to reduce the burden and control the impact of modifiable risk factors for suboptimal health status in mothers and children in suburb communities in South of Iran.

## Ethical considerations

Ethical issues (Including plagiarism, informed consent, misconduct, data fabrication and/or falsification, double publication and/or submission, redundancy, etc.) have been completely observed by the authors.
